# HLA-G Expressing Immune Cells in Immune Mediated Diseases

**DOI:** 10.3389/fimmu.2020.01613

**Published:** 2020-08-28

**Authors:** P. Contini, Giuseppe Murdaca, Francesco Puppo, Simone Negrini

**Affiliations:** Department of Internal Medicine, University of Genoa, Genoa, Italy

**Keywords:** HLA-G, immune mediated diseases, lymphocytes, dendritic cells, monocytes, NK cells, regulatory cells

## Abstract

HLA-G is a HLA class Ib antigen that possesses immunomodulatory properties. HLA-G-expressing CD4+ and CD8+ T lymphocytes, NK cells, monocytes, and dendritic cells with immunoregulatory functions are present in small percentages of patients with physiologic conditions. Quantitative and qualitative derangements of HLA-G+ immune cells have been detected in several conditions in which the immune system plays an important role, such as infectious, neoplastic, and autoimmune diseases as well as in complications from transplants and pregnancy. These observations strongly support the hypothesis that HLA-G+ immune cells may be implicated in the complex mechanisms underlying the pathogenesis of these disorders.

## Introduction

HLA-G is a HLA class Ib antigen characterized by a restricted tissue expression, low polymorphism and seven isoforms (HLA-G1 to HLA-G7) (1, 2). In both membrane-bound and soluble form, HLA-G exerts several immune-modulatory effects. It inhibits allogeneic proliferation of CD4+ T cells (3), natural killer (NK) and CD8+ T cells cytotoxicity (4), maturation of dendritic cells (DC) (5), and activation of B cells (6). In addition, soluble HLA-G molecules (sHLA-G) are able to trigger apoptosis in antigen specific CD8+ T lymphocytes (4, 7, 8).

HLA-G also seems to be involved in the tuning of immune responses. The incubation of peripheral blood mononuclear cells (PBMC) with HLA-G-expressing cells, favors a shift toward a Th-2 cytokine profile; whereas incubation with sHLA-G may have a counterbalancing effect, creating an anti-inflammatory environment due to the release of interleukin (IL)-10 (9, 10). Based on these findings, it has been recently proposed that HLA-G should be categorized as an “immune checkpoint” molecule (2).

## HLA-G+ Immune Cells in Physiologic Conditions

### T and NK Cells

Immune tolerance is based on a complex series of mechanisms that ultimately facilitate the elimination of foreign antigens, preventing collateral damage to host tissues. Immune tolerance is broadly classified into central and peripheral tolerance. Central tolerance occurs during lymphocyte development in the primary lymphoid organs, namely thymus (T cells) and bone marrow (B cells). Peripheral tolerance takes place in the peripheral tissues and lymph nodes, and consists of different immunologic mechanisms capable of controlling self-reactive lymphocytes that have escaped from central deletion (11). Immune regulation is crucial in the maintenance of peripheral tolerance and is mediated by the action of T regulatory (Treg) lymphocytes (12). Several subsets of Treg lymphocytes with distinct phenotypes and mechanisms of action have been described within both CD4+ and CD8+ T lymphocytes, and it has been clearly demonstrated that these cells play an important role in physiological and pathological conditions such as autoimmune, infectious, or neoplastic diseases (12, 13).

In 2007 Feger et al. described novel subsets of T cells that express the immunomodulatory molecule HLA-G, identifying them as distinct subpopulations of Treg lymphocytes (14). In recent years, many other studies have confirmed the importance of HLA-G+ Treg lymphocytes in physiology and disease. Similar to the classical CD4+CD25+FoxP3+Tregs, human HLA-G+ Treg cells originate from thymus and are present in variable percentages in the peripheral blood of healthy subjects (0.1–8.3%). However, HLA-G+ Treg cells can be differentiated from classical CD4+ Tregs on the basis of their distinctive phenotype, lacking Forkhead Box P3 (FoxP3), CD39, and CD25 expression (14). CD4+HLA-G+T cells have a low proliferative capacity that, differently from classic Tregs, cannot be overcome by the addition of exogenous IL-2 (14). *In vitro*, CD4+HLA-G+ Tregs inhibit T-cell responses mainly through cell-to-cell contact and independent mechanisms (15); whereas classical CD4+CD25+FoxP3+ Tregs exert their suppressive function mainly through cell-to-cell dependent mechanisms (13, 16). In both Tregs subpopulations suppressive activity depends on an optimal TCR stimulation. CD4+HLA-G+ Tregs and classical CD4+CD25+FoxP3+ Tregs share common intracellular down-stream signaling events, following T cell receptor (TCR) ligation (17, 18). They show altered activation of the linker in activation of T cells (LAT) molecules involved in proximal TCR signaling, leading to reduced intracellular calcium influx when compared to non-regulatory T-cells (16). CD4+HLA-G+ Treg cells seem to exert their suppressive function via the secretion of various tolerogenic molecules such as sHLA-G5, IL-10, IL-35, and transforming growth factor (TGF)-β (15, 16). In this context, IL-10 and sHLA-G5 are the most important molecules responsible for the immunoregulatory activity of the CD4+HLA-G+ Treg (15, 16). Transforming growth factor-β and IL-35 do not seem to have a direct role in the immunomodulation exerted by CD4+HLA-G+ Treg, nevertheless, these cytokines may indirectly promote an immunosuppressive milieu, influencing the local differentiation of peripherally induced Tregs and/or supporting the survival of thymus-derived natural Tregs (19–21).

*In vitro*, CD4+HLA-G+ Tregs display a less efficient suppressive activity than classical CD4^+^CD25^+^FoxP3^+^ Tregs; whereas *in vivo* the immunosuppressive capacity of the two Treg subsets is comparable (16). This notion suggests that CD4+HLA-G+ Tregs may modulate tissue inflammation within the target organs, in close proximity to effector T cells (16, 22).

Besides thymus-derived HLA-G+ Tregs, some normal resting and activated CD4^+^ and CD8^+^ T cells may acquire through trogocytosis the HLA-G1 molecule from antigen presenting cells (APCs), thus changing their function from effectors to regulatory cells capable of inhibiting alloproliferative responses (23). Interestingly, the acquisition of HLA-G via trogocytosis has also been described for monocytes and NK cells (24, 25). A non-cytolytic subset of HLA-G+ NK cells (NK-ireg) can be generated *in vitro* from peripheral blood CD34+ hematopoietic progenitors expressing membrane-bound IL-15. NK-ireg cells display a mature NK cell phenotype, release suppressive molecules (HLA-G, IL-10, and IL-21), and through these factors are capable of suppressing the cytotoxicity of DC and NK cells (26).

It has been recently reported that neutrophil gelatinase-associated lipocalin seems to be capable of upregulating HLA-G expression and expansion of Tregs cells in healthy donors (27). This observation is consistent with the knowledge that lipocalin family members act as modulators of many different physiological and pathologic processes, including cell differentiation, proliferation and apoptosis (28). Moreover, HLA-G expression is strongly regulated by methylation, and it has been recently observed that hypomethylating agents such as azacytidine and decitabine, can induce *de novo* expression of HLA-G on conventional T cells thus converting the latter into HLA-G+ Tregs (29). This data suggest the possibility of modulating the expansion of HLA-G-expressing T cells *in vivo* or generating them *in vitro* for adoptive immunotherapy in transplant patients or for other immunological disorders.

### Monocytes

The expression of HLA-G in human mononuclear phagocytes and APC has been known for many years (30, 31). HLA-G cell surface expression has been detected at variable percentages in peripheral blood CD14+ monocytes from healthy individuals (32–36). HLA-G mRNA and intracellular HLA-G levels as well as surface HLA-G expression are selectively increased after *in vitro* treatment of monocytes with interferon (IFN)-β, IFN-γ, and IL-10 (30, 32).

As far as the functional role of CD14+HLA-G+ cells is concerned, it has been reported that they have limited *in vitro* immunostimulatory function and are able to inhibit T-cell alloproliferation when added in mixed lymphocyte cultures. The suppressive function of CD14+HLA-G+ cells is related to the expression of the HLA-G molecule, which can be antagonized by blocking HLA-G with specific monoclonal antibodies, and may also be mediated through sHLA-G, as suggested by transwell experiments. Further *in vitro* experiments have shown that co-incubation of CD4+ and CD8+ T cells with CD14+HLA-G+ cells decreases the surface expression of CD4 and CD8 molecules and inhibits both Th1 and Th2 cytokine production by antigen-stimulated autologous CD4^+^ T cells (37, 38).

Monocytes can differentiate into a range of functional subsets including pro-inflammatory (M1) and anti-inflammatory (M2) cells. Recently published data indicates that M2 cells obtained from peripheral blood monocytes after *in vitro* activation with IL-4, express high amounts of HLA-G and drive upregulation of the HLA-G ligand immunoglobulin-like transcript (ILT)-2 on NK cells. This leads to the generation of hyporesponsive CD56^dim^ NK cells with limited degranulation and cytotoxic activity (39).

### Dendritic Cells

Peripheral blood DCs are APCs that regulate innate and adaptive immune responses. Different DC subsets have been identified that can drive immune responses toward immunity or tolerance, including conventional monocytoid DCs that maintain immunological homeostasis and can induce tolerance, plasmacytoid DCs that present foreign antigens, activate Tregs, and tolerogenic DCs which promote tolerance.

The expression of HLA-G on DC may be regulated by cytokines. *In vitro* experiments have shown that TGF-β increases HLA-G expression by DC and that HLA-G+ DC down-regulate activation of CD4+ T cells and production of IL-6 and IL-17, suggesting the possibility that HLA-G+ DC plays a role in immunoregulatory *in vivo* (40).

Recently, a subset of human DC has been characterized. Termed DC-10, these human DC have the ability to secrete IL-10. DC-10 are found in peripheral blood and the spleen of healthy individuals. They can be generated *in vitro* by culturing peripheral monocytes in the presence of IL-10. Furthermore, DC-10 are highly represented in the decidua of pregnant women when compared to peripheral blood, suggesting that these cells may accumulate at the fetal maternal interface to promote tolerance to the semi-allogeneic fetus (41). DC-10 have a mature phenotype and express CD11c, CD14, CD16, CD141, and CD163. DC-10 also express HLA-G and ILT-4 and are able to induce T regulatory type 1 (Tr1) cells. The amount of HLA-G expression on DC-10 is genetically driven and is associated with specific variations in the 3’ untranslated region of the HLA-G gene. Of particular interest are findings on the capacity of DC-10 to induce Tr1 cells, which correlates with the level of HLA-G expression. These data indicate that HLA-G expression plays a fundamental role in the tolerogenic activity of DC-10 and suggest a potential clinical use of DC-10 as an immunomodulatory treatment (42–44).

Collectively, results of *in vitro* and *in vivo* experiments indicate that HLA-G positive DC can affect the activity of NK cells, modulate the response of effector CD4+ and CD8+ T cells, and induce Tregs. These findings strongly support the notion that HLA-G expression by DC plays a central role modulating innate and adaptive immune responses in a healthy state and in pathological conditions.

## HLA-G-Expressing Immune Cells in Non-Autoimmune Diseases

### T and NK Cells

Lymphocytes expressing HLA-G have been reported in several diseases in which the immune system plays a pivotal role, such as neoplastic, infectious, and autoimmune/inflammatory disorders.

An increase in the percentage of CD8+HLA-G+ T cells and the presence of HIV-1-specific CD8+HLA-G+ T lymphocytes have been described in HIV-1 patients, although their exact pathophysiologic role in the disease is still elusive (36, 45, 46). Other authors observed that HLA-G+ Treg may reduce harmful bystander immune activation, while minimally inhibiting antiviral T cell-mediated responses, thus suggesting the positive role of these cells in the natural history of HIV infection (47).

Reduced percentages of HLA-G expressing T cells and monocytes have been observed in pre-eclamptic patients, compared with women with a healthy pregnancy or healthy control subjects (48). By analogy with these results, Hsu and colleagues reported that CD4+HLA-G+ T cells are significantly expanded in the peripheral blood of pregnant women compared with non-pregnant controls and pre-eclamptic women (49). In addition, CD4+HLA-G+ T cells tend to accumulate in the decidua of healthy pregnant women; whereas this phenomenon is impaired in pre-eclamptic patients (41). A small subset of NKp46+HLA-G+IL-10+ NK cells has been described *in vivo* among the decidual NK cells of pregnant women, but the exact role of this cell subset requires further investigation (26).

Recently, a novel population of CD4^low^HLA-G+ T cells, identified as IL-4-expressing Th17 cells, has been described in prostate cancer and their expansion seems to correlate with the increase of tumor aggressiveness (50). Increased percentages of HLA-G+CD3+ cells have been observed in the peripheral blood of breast cancer patients, suggesting that these cells may contribute to tumor development by down-modulating antitumor immunity (51). Moreover, it has also been reported that a subset of HLA-G+ NK cells possessing suppressive activity are considerably increased in the peripheral blood of breast cancer patients (52).

It is well-known that, in order to escape immune-surveillance, various malignant cells can aberrantly express HLA-G and/or secrete sHLA-G (53, 54). In addition, cancer cells can induce HLA-G-expressing immune cells (e.g., transferring HLA-G to T cells through trogocytosis) within the tumor microenvironment. This mechanism may increase the number of local immunosuppressive cells, thus facilitating tumor immune-escape (23, 55, 56).

A similar mechanism of immune-evasion has also been described for microbial infections, in fact *Pseudomonas aeruginosa* seems to be capable, at least *in vitro*, of inducing HLA-G expression in immune cells, creating a protected niche and facilitating bacterial survival (57).

In the context of kidney transplants, it has been reported that HLA-G expression on T cells increases after the transplant, but significantly decreases in subjects experiencing an acute rejection. This data suggests that HLA-G might be involved in the protection of transplants against rejection and the levels of HLA-G on CD4+ may represent a potential marker in predicting episodes of renal rejection after kidney transplantation (58, 59).

In patients that experience an allergic reaction, HLA-G expression as well as sHLA-G secretion are increased in CD4+ cells and monocytes after *in vitro* stimulation by the causal allergen, but not by non-specific stimuli and non-causal allergens (60). This data suggest that HLA-G may be involved into the pathogenetic mechanisms underlying allergic inflammation and allergen specific immunotherapy (60, 61).

### Monocytes and Dendritic Cells

Several *in vivo* data-sets support the immunomodulatory properties of HLA-G+ monocytes. In fact, a high frequency of CD14+HLA-G+ cells have been detected in patients undergoing allogeneic hematopoietic cell transplantation. These HLA-G+ monocytes appear early post-transplant and remain at high levels for up to one year after the transplant. It is of interest that HLA-G+ monocytes have also been detected in skin biopsies of transplanted patients who developed graft-*versus*-host disease. It may be hypothesized that the increase of HLA-G+ monocytes could be related to an alloreaction occurring after transplant (37).

Elevated numbers of HLA-G+ monocytes have been found in the peripheral blood of HIV-1-infected individuals. The expression of HLA-G might either be directly caused by the HIV-1 virus infection or indirectly related to increased levels of IL-10, which is known to induce HLA-G expression in monocytes. By decreasing the antigen-presenting capacity of monocyte, the upregulated expression of HLA-G could be one of the strategies used by the HIV-1 virus to evade immune surveillance (36).

Collectively, *in vitro* and *in vivo* data suggests that monocyte activation by cytokines, infectious agents, and allogeneic stimuli induces HLA-G expression. Taking this into account, HLA-G+ monocytes may exert immunosuppressive effects on CD4+, CD8+, and NK cells, playing a role in down-regulation of the immune response.

Concerning DCs, it has been reported that monocytoid DC expressing high HLA-G levels can be found in the peripheral blood of stable and tolerant liver transplant recipients. The number of HLA-G+ DC correlates with the percentage of CD4+CD25^high^CD127- Tregs and with the intensity of Foxp3 expression, thus supporting the hypothesis that HLA-G+ DC may play a tolerogenic role in alloimmune reactivity (62).

### Mast Cells

Mast cells are bone marrow derived cells that circulate in an immature form and become mature after migration in a tissue site. Mast cells have been mostly viewed as effectors of IgE-mediated allergic diseases and host defense against parasites. The role of mast cells in both innate and adaptive immunity has been recognized recently. In addition, mast cells are involved in tissue repair through the secretion of several cytokines and growth factors that enhance fibroblast proliferation and collagen deposition, and inhibit degradation of the extra cellular matrix (63).

To our present knowledge it is not known whether mast cells express HLA-G in physiological conditions, and emerging research on the role for HLA-G+ mast cells in liver diseases is of interest (64, 65). It has been reported that mast cells infiltrating the livers of patients infected with hepatitis C virus (HCV) express HLA-G and secrete HLA-G in soluble form. The number of HLA-G+ mast cells is significantly associated with the areas of connective tissue and liver fibrosis located close to the hepatic arteries, veins and bile ducts of the portal tracts (66, 67). The presence of mast cells in the liver can be related to the production of TGF-β, a potent mast cell chemoattractant, by hepatic stellate cells (HSC)(68, 69). Then, HLA-G+ infiltrating mast cells promote HSC proliferation that, in turn, induces liver fibrosis (70). The expression and secretion of HLA-G by mast cells in HCV infected patients can be explained by the elevated amounts of IFN-α and IL-10 produced during HCV infection (71, 72). Accordingly, *in vitro* and *in vivo* data indicate that these cytokines strongly modulate HLA-G up-regulation in monocytes and other cells including trophoblasts, fibroblasts, and neoplastic cells (32, 73–78). The function of HLA-G+ mast cells during HCV infection remains to be clarified. It may be suggested that HLA-G expression may promote viral escape from the immune system by inhibiting both adaptive and innate immunity, thus protecting HCV-infected cells and favoring viral progression.

In summary, this data supports the assumption that HLA-G+ immune cells are implicated in the pathogenesis of a wide array of disorders. The role of HLA-G+ immune cells in the context of autoimmune diseases will be discussed in the following paragraphs.

## HLA-G-Expressing Immune Cells in Autoimmune Diseases

### Multiple Sclerosis

Multiple sclerosis (MS) is an immune-mediated disorder of the central nervous system (CNS) leading to demyelination as well as axonal and neuronal damage, with progressive neurological impairment (79). The course of MS can follow four clinical patterns that include relapsing remitting MS (RRMS, which accounts for 80–90% of MS cases at onset), secondary progressive MS (SPMS), primary progressive MS (PPMS), and progressive relapsing MS (PRMS) (80). Although the pathogenesis of MS is still not completely understood, it is known that central tolerance may be defective leading to the development of self-reactive T cells that transmigrate into the CNS where they can be activated by APCs and determine brain damage (79). The brain has long been considered an immunologically privileged site. This idea is based on the observation that tissue transplants in the CNS are not commonly rejected by the immune system. Commonly accepted explanations for the lack of an effective immune response to antigens in the brain are an anti-inflammatory and, with regard to invading immune cells, pro-apoptotic environment in the brain, the limited access of brain-derived antigens to the lymphoid organs, the presence of the blood–brain barrier, low major histocompatibility complex (MHC) expression in the brain parenchyma, and the absence of DCs (81, 82). However, numerous studies in infectious, autoimmune and tumor models have challenged this view by showing that potent immune reactions can and do occur in the CNS (83).

The main aspect favoring the autoimmune etiology of MS consists of the presence of activated IFN-producing T helper 1 (Th1) cells, that recognize peptides of the myelin sheath, including myelin basic protein (MBP), proteolipid protein (PLP), and myelin oligodendrocyte glycoprotein (MOG) (80). HLA-G immunoreactivity was detected in the transition zone between the plaque center and the perilesional areas as well as in both acute and chronic active plaques. In proximity of MS lesions the adjacent normal appearing gray matter remained predominantly negative for HLA-G, whereas HLA-G expression in adjacent normal appearing white matter was similar to the expression levels of the lesion borders (83). Notably, in early and highly inflammatory MS lesions, HLA-G expression was abundant and detected on macrophages/activated microglia cells. Similarly, perilesional activated microglia cells were immunoreactive for HLA-G. Furthermore, endothelial cells and meningeal vessels as well as arachnoidal cap cells show HLA-G immunoreactivity (83). However, expression of the inhibitory receptors for HLA-G, belonging to immunoglobulin-like transcript family ILT2 and ILT4, have been described in chronic active MS plaques. ILT2 immunoreactivity could be observed in the plaque center and the plaque border and paralleled HLA-G immunoreactivity. The main cellular sources for both molecules were macrophages and microglia (83). The cerebrospinal fluid (CSF) compartment has been proposed to partially constitute a functional equivalent of the lymphatic system for the CNS. Interestingly, the levels of HLA-G on CD14^+^ monocytes were significantly elevated in the CSF of patients with MS compared with peripheral blood. Of note is the fact that HLA-G expressed by monocytes was identified as an important negative immune-regulatory factor, down-regulating the production of Th1 as well as Th2 cytokines, inhibiting antigen-specific and autologous CD4^+^ T-cell activation, and inducing anergic T cells (37, 38). Furthermore, a small number of both CD4^+^ and CD8^+^ T cells, including CD4^+^ Tregs, expressed HLA-G in the CSF of MS patients (83). Interestingly, CSF-derived HLA-G+CD4+ Tregs show high expression of the C-C chemokine receptor 5 that might favor their selective migration into the nervous system of MS patients, counteracting the activity of autoreactive T cells. The frequency of CSF-derived HLA-G+CD4+ Tregs correlates positively with the disease status in MS patients with active disease (22). Increased levels of HLA-G+CD4+ Tregs have been detected in MS patients responses to IFN or natalizumab treatment (80).

Taken together, these findings, seem to confirm that HLA-G expression on immune cells infiltrating CNS and detectable in CSF, may contribute to immune-regulation in MS.

### Systemic Lupus Erythematosus

Systemic lupus erythematosus (SLE) is an autoimmune inflammatory disease that can affect virtually any organ system, including skin, joints, kidneys, brain, and blood vessels (84). The development of SLE is dependent on a complex interplay between genetic, environmental, and immunological factors (85, 86). Among these, defective function of regulatory T cells and polyclonal activation of B lymphocytes leading to the production of auto-antibodies seems to play a major role (87–89).

Limited literature data are available on the expression of HLA-G in immune cells from SLE patients. Monsivais-Urenda et al. reported that monocytes from SLE patients as well as mature CD83^+^ DC showed a reduced expression of HLA-G compared with healthy controls (35). In addition, monocytes from SLE patients showed a diminished induction of HLA-G expression in response to stimulation with IL-10, and when pre-treated with IFN-γ they exhibited an impaired capability to inhibit the proliferation of autologous lymphocytes. Interestingly, lymphocytes from SLE patients seem to display a lower capability to acquire HLA-G molecules by trogocytosis from autologous monocytes as compared to lymphocytes from normal subjects (35). By contrast, our and other groups reported that the percentage of HLA-G expressing cells among PBMC is significantly higher in SLE patients than in healthy controls (33, 90). In particular, the percentages of HLA-G-positive monocytes and HLA-G-expressing CD4+, CD8+, and CD4+/CD8+ double positive (DP) cells are significantly higher in SLE patients than in controls. Moreover, within the population of DP cells a subpopulation of CD4^dull^CD8^high^ cells displayed a high proportion of HLA-G^+^ cells, while HLA-G was virtually absent in the same subpopulation of healthy subjects (33). The function of circulating HLA-G+ DP cells is not known, however, it is worth noting that DP cells seem to exert a suppressive role in the production of autoantibodies in SLE patients (91). In summary, it may be proposed that the up-regulation of HLA-G membrane expression by PBMC could reflect an effort to regulate the hyperactive immune status occurring in SLE.

### Systemic Sclerosis

Systemic sclerosis (SSc) is a chronic connective tissue disease of unknown origin, more frequently affecting women. It is characterized by diffuse fibrosis, vasculopathy and immune dysregulation. In addition to skin involvement, SSc can affect multiple organ systems, including the musculo-skeletal, pulmonary, cardiac, gastrointestinal, and urinary systems (92, 93). Complex alterations of the normal functional balance within immune cells sub-populations, in particular Th17 lymphocytes and Tregs, including both CD4+ and CD8+ Tregs subsets, have been demonstrated in patients affected by SSc (94–97).

Our research group analyzed the role of both membrane HLA-G and sHLA-G in SSc patients. In particular, we recently reported that the percentage of HLA-G-positive monocytes, CD4+ T cells, CD8+ T cells and DP cells are significantly higher in SSc patients as compared to healthy subjects (34). Among DP cells a subpopulation of CD4^*dull*^CD8^high^ lymphocytes highly expressing HLA-G was detected. The function of circulating DP cells in SSc is under investigation, however, it is worth noting that these cells, which may exert potent suppressive effects, are present in the inflamed tissues of patients affected by immune mediated disorders and in the skin of patients with early active SSc. This may contribute, through IL-4 secretion, to the enhanced extracellular matrix deposition by fibroblasts (98). Plasma sHLA-G levels were higher in SSc patients when compared to healthy controls. Notably, plasma levels of sHLA-G1 and sHLA-G5 isoforms were comparable and no significant differences were detected in total sHLA-G, sHLA-G1 and sHLA-G5 levels between limited and diffuse SSc forms. The total sHLA-G plasma levels correlated with the elevated TGF-β levels circulating in SSc patients (34). This finding is in agreement with *in vitro* data demonstrating that the production of TGF-β by myelomonocytic cells is strongly increased after incubation with recombinant sHLA-G (99). In summary, it may be proposed that there is a possible involvement of HLA−G in SSc pathogenesis, as the elevated HLA−G membrane expression by PBMC and the increased sHLA−G plasma levels may reflect an attempt to control the immune derangement occurring in this disease and concur, through TGF-β up-regulation, with fibroblast activation and fibrosis development (34).

### Skin Diseases

Psoriasis (Ps) is a common inflammatory, chronic, and disabling skin disease that affects 1–3% of the population (100). Distinct clinical phenotypes may be observed in this disease, including chronic plaque (Ps vulgaris), guttate, and pustular variants. At least 10% of patients can develop arthritis (101). In many cases, a marked infiltration of mononuclear leucocytes (T lymphocytes and DC) into the dermis and elongated/hyperplastic blood vessels in the papillary dermal region can be observed (102). Because Ps is considered to be an organ-specific autoimmune disease, Cardili et al. analyzed HLA-G expression in skin specimens obtained from patients with Ps and observed the presence of HLA-G molecules on lymphomononuclear cells within the dermis and to a higher extent, in the epidermis. The intensity of HLA-G expression was not correlated with Ps variants or severity. By contrast, skin specimens obtained from healthy individuals were negative for HLA-G expression (103). Other authors have reported HLA-G expression in CD68^+^ CD11c^+^ macrophages lining the dermo-epidermal junction in patients with Ps vulgaris (104). In addition, NK cells and CD4^+^ T cells expressing the IL2 inhibitory receptor have been described in Ps skin infiltrates, suggesting that HLA-G may act as an inhibitory molecule to down-regulate the activation of effector cells (104). These findings lead to the assumption that HLA-G^+^ macrophages could represent an internal control system that counteracts auto-reactive expression of T-cell cognate receptors for HLA-G.

Atopic dermatitis (AD) is another chronic T-cell mediated skin disorder which, in contrast to Ps, exhibits a Th2 type cytokine profile including over-production of IL-10, which is known to up-regulate HLA-G (105). Khosrotehrani et al. investigated the role of HLA-G in patients with AD. They found that HLA-G was mainly expressed by infiltrating T cells and to a lesser extent, by macrophages and even DC (106). The epidermis was consistently negative for HLA-G expression, suggesting that, analogously to Ps, HLA-G up-regulation may either be the consequence of the permissive cytokine environment in AD or it may be a part of an internal regulatory system to control excessive inflammation.

## Discussion

Data in recent literature indicates that small percentages of HLA-G positive immune cells can be detected in the peripheral blood of patients with physiological conditions. In these conditions HLA-G positive immune cells seem to play an emerging role in maintaining immune homeostasis. However, increased percentages of circulating and tissue infiltrating HLA-G positive immune cells occur in various pathological conditions like infections, cancers, transplants, and immune-mediated diseases. Taking into account the immunoregulatory role of HLA-G, it may be suggested that T lymphocytes, NK cells and APCs that express HLA-G molecules are potentially involved in the pathogenesis of immune mediated diseases.

HLA-G positive cells can modulate both the priming and the effector phases of the immune response, thus contributing to peripheral immune tolerance. It may be proposed that HLA-G expressing immune cells represent an attempt to create an immune-suppressive milieu, as a way of controlling immune derangement in systemic autoimmune disorders. However, several important issues still need to be clarified in this context. A better understanding of the HLA-G gene regulation will greatly improve the possibility of manipulating this emerging immune check-point, which could alter the course of immunological diseases. Moreover, the role played by different molecular HLA-G isoforms and the contribution of specific HLA-G expressing subpopulations in each clinical situation needs to be better defined. Therefore further pre-clinical and clinical investigations are required in order to provide more detailed information on the role played by HLA-G expressing cells in the mechanisms underlying the onset and progression of immune-mediated diseases. These future studies are crucial for the development of potential HLA-G strategies of therapy.

## Author Contributions

All authors equally contributed to the conception of ideas and design of this manuscript.

## Conflict of Interest

The authors declare that the research was conducted in the absence of any commercial or financial relationships that could be construed as a potential conflict of interest.
